# Perspectives on virtual interviews and emerging technologies integration in family medicine residency programs: a cross-sectional survey study

**DOI:** 10.1186/s12909-024-05874-5

**Published:** 2024-09-09

**Authors:** Raymond Tolentino, Charo Rodriguez, Fanny Hersson-Edery, Julie Lane, Samira Abbasgholizadeh Rahimi

**Affiliations:** 1 Department of Family Medicine, School of Medicine, Faculty of Medicine and Health Sciences, McGill University, Montréal, Canada; 2 Institute of Health Sciences Education, Faculty of Medicine and Health Sciences, McGill University, Montreal, Canada; 3 Lady Davis Institute for Medical Research, Jewish General Hospital, Montréal, Canada; 4 Mila - Quebec AI Institute, Montreal, Canada; 5 Faculty of Dental Medicine and Oral Health Sciences, McGill University, Montreal, Canada

**Keywords:** Medical residency, Family medicine, Virtual interview, Artificial intelligence, Virtual reality

## Abstract

**Background:**

During the coronavirus disease of 2019 (COVID-19) pandemic, in-person interviews for the recruitment of family medicine residents shifted to online (virtual) interviews. The purpose of this study was twofold: (1) to gather the ideas about virtual interviews of family medicine applicants (interviewees), and faculty and staff who interviewed these applicants (interviewers), and (2) to describe interviewers’ and interviewees’ opinions of use of emerging technologies such as artificial intelligence (AI) and virtual reality (VR) in the recruitment process as well as during clinical practice.

**Methods:**

This was a cross-sectional survey study. Participants were both interviewers and candidates who applied to the McGill University Family Medicine Residency Program for the 2020–2021 and 2021–2022 cycles.

**Results:**

The study population was constituted by *N* = 132 applicants and *N* = 60 interviewers. The response rate was 91.7% (55/60) for interviewers and 43.2% (57/132) for interviewees. Both interviewers (43.7%) and interviewees (68.5%) were satisfied with connecting through virtual interviews. Interviewers (43.75%) and interviewees (55.5%) would prefer for both options to be available. Both interviewers (50%) and interviewees (72%) were interested in emerging technologies. Almost all interviewees (95.8%) were interested in learning about AI and VR and its application in clinical practice with the majority (60.8%) agreeing that it should be taught within medical training.

**Conclusion:**

Although experience of virtual interviewing during the COVID-19 pandemic has been positive for both interviewees and interviewers, the findings of this study suggest that it will be unlikely that virtual interviews completely replace in-person interviews for selecting candidates for family medicine residency programs in the long term as participants value aspects of in-person interviews and would want a choice in format. Since incoming family medicine physicians seem to be eager to learn and utilize emerging technologies such as AI and VR, educators and institutions should consider family physicians’ needs due to the changing technological landscape in family medicine education.

**Supplementary Information:**

The online version contains supplementary material available at 10.1186/s12909-024-05874-5.

## Background

Due to the coronavirus disease of 2019 (COVID-19) pandemic, there have been several disruptions within medical education, one of these being medical residency recruitment [1–3]. In effect, during this period, the recruitment and evaluation of residency applicants had to rapidly move from the traditional in-person interviews to virtual interviews [4]. Prior to COVID-19, residency applicants would travel for in-person interviews, with only a small number of programs offering virtual interviews. During on-site visits, applicants would meet with the program faculty and leadership, current residents as well as visit the teaching units and other healthcare facilities [5]. The in-person process had many benefits for applicants including learning about the culture, the program, and the location – important factors that contribute to an applicant’s decision process [6]. However, due to the concerns surrounding travel during the COVID-19 pandemic, the Association of Faculties of Medicine of Canada (AFMC) Board of Directors decided that resident match interviews had to be conducted in a virtual format [7, 8]. In response to this recommendation, McGill University Family Medicine, as other Family Medicine academic units in the country and elsewhere, has conducted residency application cycle virtually since 2020.

There are several advantages of utilizing virtual interviews, e.g., financial savings for both applicants and postgraduate faculty, decreased travel time, and reduced environmental impact as well as transmission of COVID-19 [3]. However, virtual interviews can create new obstacles, for instance possibility of technical challenges, lack of personal connection between applicants and faculty, decreased interaction between applicants and current trainees, decreased opportunity to attend informal gatherings, and finally an increased difficulty for the applicant to view the culture of the program, the hospital campus, and the city in-person [3].

Videoconferencing platforms for residency recruitment constitute one of the information technologies whose use has skyrocketed everywhere during the COVID-19 pandemic [15]. Emerging technologies such as artificial intelligence (AI) and virtual reality (VR) have also been expanding within the medical field, as seen in clinical practice, recruitment of residents, and education [16–18]. For example, AI can analyze complex medical data in clinical practice [16] and provide feedback and assessment during training [18], while VR simulations can be used for hospital tours during residency recruitment [17]. As technological innovations continue to grow in all health care facilities (and not only in hospital settings), but it is also important to understand how family physicians perceive these technologies, as well as how trustable and reliable they consider they are in family medicine education and practice.

There is no doubt that the virtual format will change the processes whereby applicants learn about prospective programs and how faculty interviewers will learn about potential applicants. To navigate this new landscape, several studies and medical school programs have provided tips and preparation techniques [3, 9, 10]. Nowadays, it remains unclear whether travelling to residency interviews will continue. Therefore, it is important to improve the virtual interview process to provide the best experience as well as match outcome for both residents and program. Several studies have been published since COVID-19, which have detailed the change from conducting interviews in-person to virtual interviews for residency selection. These studies come from different residency programs such as radiology, neurosurgery, internal medicine, pediatrics, and otolaryngology [1, 5, 9–14]. In general, these works demonstrate candidates’ positive subjective experience with virtual interviews. However, the inability to get to know the program and if they are the right fit to it have been some of the deleterious effects also reported [12].

In this context, and to the best of our knowledge, few studies have described the experience of applicants and residency program directors of using virtual interviews for entering in a family medicine postgraduate residency program. We therefore decided to undertake an empirical investigation guided by the following research questions: (1) What are successful applicants’ and faculty members’ opinions about their experience of virtual interviews for entering in a family medicine postgraduate residency program (2)? What are their views about the use of emerging technologies such as AI and VR in the medical residency matching process as well as in clinical practice?

## Methods

### Study design

We conducted a cross-sectional survey study. The survey followed the Checklist for Reporting Of Survey Studies (CROSS) guidelines to guide the reporting of the results [19]. Moreover, it adhered to the tenets of the Declaration of Helsinki [20]. Ethics approval was obtained from the Faculty of Medicine and Health Sciences Institutional Review Board at McGill University (#A01-B09-22 A).

### Study population and sample characteristics

We included two groups of participants: (1) all family medicine residency applicants (interviewees) and (2) family medicine faculty and staff (interviewers) who interviewed family medicine residency applicants. Interviewees must have participated in a virtual interview at McGill University in the 2020–2021 and 2021–2022 cycles. Interviewers were faculty members at Department of Family Medicine, School of Medicine, Faculty of Medicine and Health Sciences, McGill University. The total number of interviewees for the 2020–2021 and 2021–2022 cycles were 192 while the total number of interviewers were 60 for both cycles. All interviewees and interviewers were recruited for participation from 16 March 2022 to 30 April 2022.

### Data collection

The survey was constructed using an online web survey platform, Lime Survey (Version 3.24.2). Two different types of surveys were constructed, one specific to the interviewee population and the other specific to the interviewer perspective. Each survey package consisted of three sections that took about 20 min to complete. The first section included sociodemographic questions such as age, gender, ethnicity, and educational background. The sociodemographic survey was identical for both groups (Appendix 1).

The second section included 11 questions (interviewer version) or seven questions (interviewee version) which focused on the overall experience of participating in online interviews (Appendix 2). The questions for this section of the survey were adapted from validated surveys used for a similar purpose in other studies [1, 12, 21]. The final section of the survey included nine questions (interviewer version) or ten questions (interviewee version) which focused on the participants’ perceptions and knowledge of emerging technologies such as AI and VR during virtual interviews as well during clinical practice (Appendix 3). Questions for these sections were in the style of 5-point Likert items, binary items, and free text responses.

Most questions were close ended while three questions were open-ended. The surveys for both interviewees and interviewers were offered both in English and French. The first author (RT) designed the electronic survey, which was first revised by the rest of co-authors, and then pilot tested with two members of each group for relevance, clarity, ease of understanding and time taken to complete. Both versions of the surveys were finally edited based on feedback and reviewed prior to email distribution.

### Survey administration

Invitations were emailed to all interviewers and interviewees on 16 March 2022 with two reminder emails sent to each group after two weeks and four weeks. The invitation email included the purpose of the study as well as a link to the consent form and survey. Individuals who accepted to participate clicked on the link where the consent form was displayed. No compensation was given to participants. To move forward in the survey, participants had to read and accept the electronic consent form by checking off a box within the survey platform. The survey was active from 16 March 2022 to 30 April 2022, and responses were recorded anonymously.

### Data analysis

All completed and partially completed questionnaires were included in the final analysis. The results from the online survey platform Lime Survey were exported to Microsoft Excel (version 16.50; Microsoft, Redmond, WA) to calculate the frequencies and percentages of each survey item. Descriptive statistics (frequency, percentage) on survey questions were displayed in a tabular or graph format. Positive choices (strongly agree and somewhat agree) and negative choices (strongly disagree and somewhat disagree) were collapsed into one. The chi-square test (x^2^) of association or Fisher’s exact test were performed, where appropriate, between participants to assess the association between categorically measured variables. The p-values of these tests were presented only, where appropriate, and statistically significant differences were considered when *p* ≤ 0.05. These statistical tests were performed on the statistical software package, R and its user interface, R Studio (Version 1.4.1717 2009–2021 RStudio, PBC). Text responses were analyzed using conventional content analysis by Hsieh & Shannon (2005) [22].

## Results

### Respondent characteristics

The survey involved 112 participants in all, comprising 57 interviewees and 55 interviewers. 48 interviewers and 54 interviewees, however, finished the entire survey. While the response rate for interviewees was somewhat lower at 29.7%, the response rate for interviewers was high at 91.7%. Among the respondents, most participants were female for both groups with 85.5% for interviewers and 66.7% for interviewees. More than 70% of interviewee participants were less than 30 years old and 43.6% of interviewers were older than 40 years old. The primary language was French for 65.5% of interviewers while it was 36.8% for interviewees. Respondents’ characteristics did not differ with non-responders’. There is no significant difference in the distribution of gender, age, and educational background between interviewees and interviewers while a difference can be seen in relation to the distribution of ethnicity, language, and educational level. Table 1 displays further participant characteristics.

**Table 1:** Demographic characteristics of participants

	Interviewers *N* = 55 *n*(%)	Interviewees *N* = 57 *n*(%)	*p*-value
**Gender**			
Male	7 (12.7)	18 (31.6)	*p* = 0.0562
Female	47 (85.5)	38 (66.7)	
Preferred Not to Answer	1 (1.8)	1 (1.7)	
**Age Group**			
≤ 25 years old	0 (0)	17 (29.8)	*p* = 0.0805
26–30 years old	5 (9.1)	25 (43.9)	
31–35 years old	15 (27.3)	11 (19.3)	
36–40 years old	11 (20)	2 (3.5)	
≥ 41 years old	24 (43.6)	2 (3.5)	
**Ethnicity**			
Caucasian	49 (89.1)	27 (47.4)	*p* = 0.00
Black of African Canadian	0	4 (7.0)	
Asian or Pacific Islander	0	11 (19.3)	
Hispanic or Latinx	0	1 (1.7)	
Multiracial or Biracial	1 (1.8)	3 (5.3)	
Indigenous	0	0 (0)	
Other	1 (1.8)	2 (3.5)	
Preferred Not to Answer	4 (7.3)	9 (15.8)	
**Primary Language**			
English	17 (30.9)	25 (43.9)	*p* = 0.0029
French	36 (65.5)	21 (36.8)	
Other	2 (3.6)	11 (19.3)	
**Highest Education Level**			
Bachelor’s Degree	13 (23.6)	16 (28.1)	*p* = 0.0001
Master’s Degree	2 (3.6)	18 (31.6)	
Doctorate Degree	33 (60)	14 (24.5)	
Other	7 (12.7)	9 (15.8)	
**Educational Background**			
General Sciences, Biomedical Sciences and Health Sciences	50 (90.9)	42 (73.7)	*p* = 0.0940
Arts and Humanities	2 (3.6)	2 (3.5)	
Business	0 (0)	2 (3.5)	
Engineering, Math and Computer Sciences	0 (0)	3 (5.3)	
Other	3 (5.5)	8 (14.0)	

### Participants’ experience with virtual interviewing

#### Interviewees

**Fig. 1:**
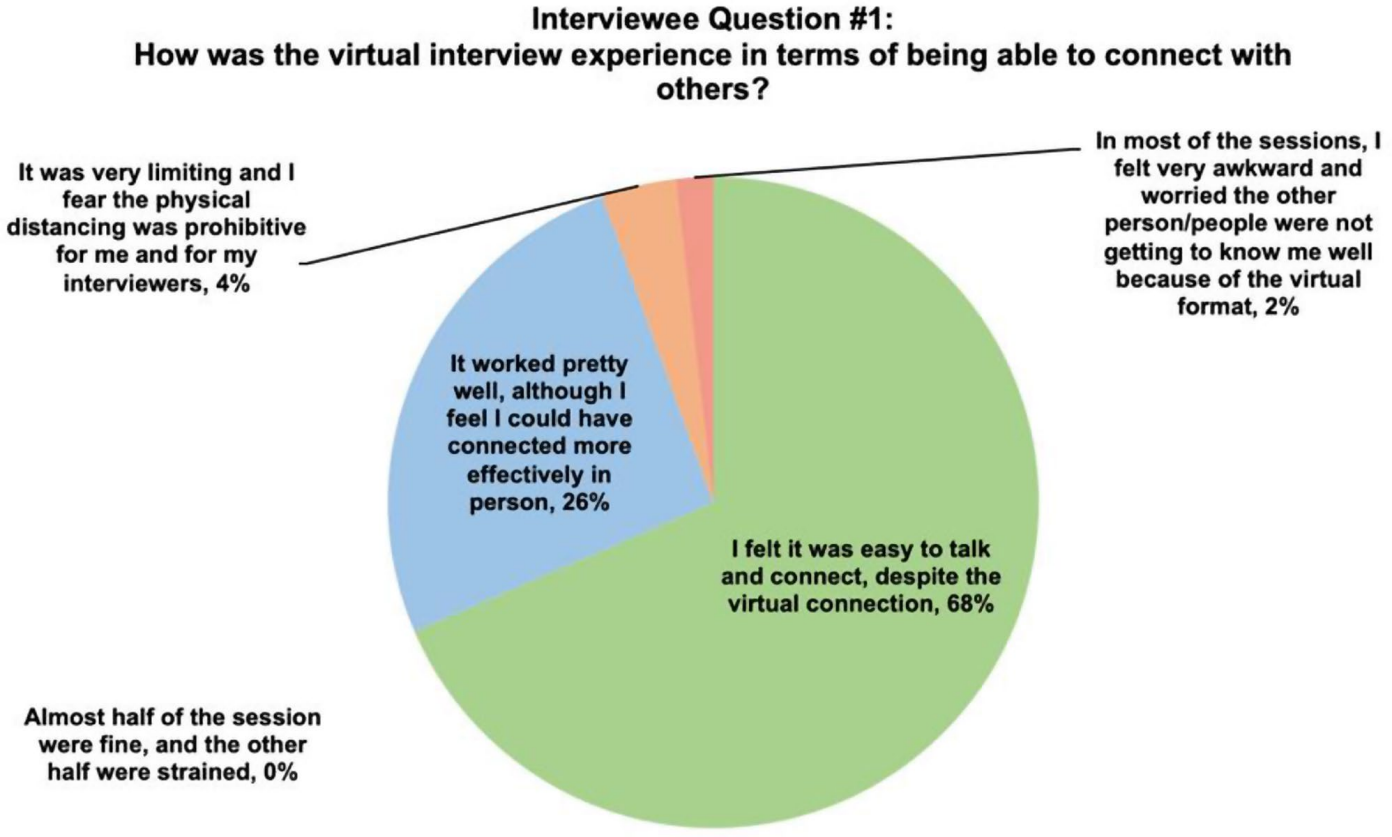
Results from interviewees on their perspective of using virtual interviews to being able to connect with others and properly interview candidates

**Fig. 2:**
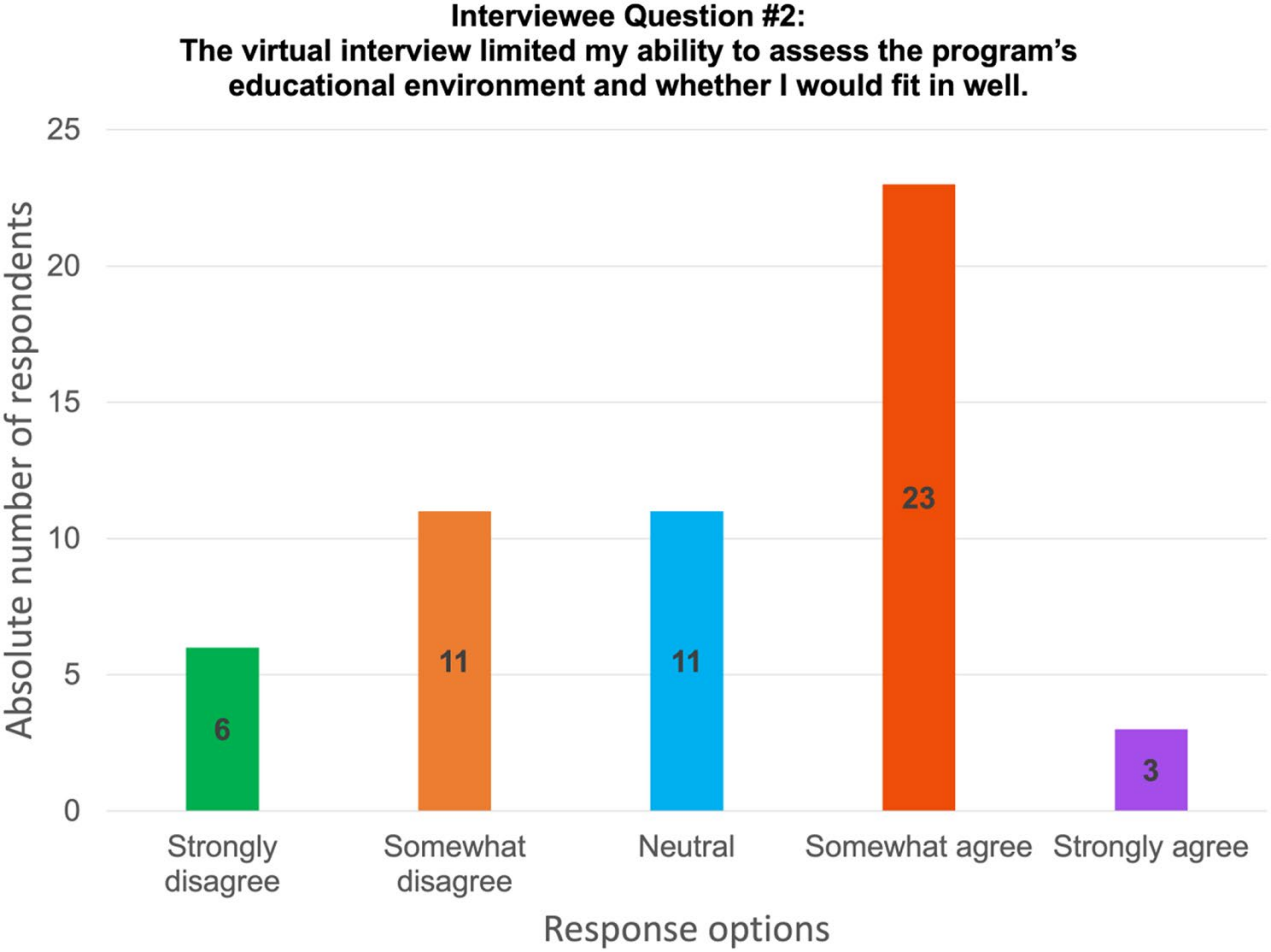
Results from interviewees on their perspective of how limiting virtual interviews were for assessing the educational environment and fit into the program

**Fig. 3:**
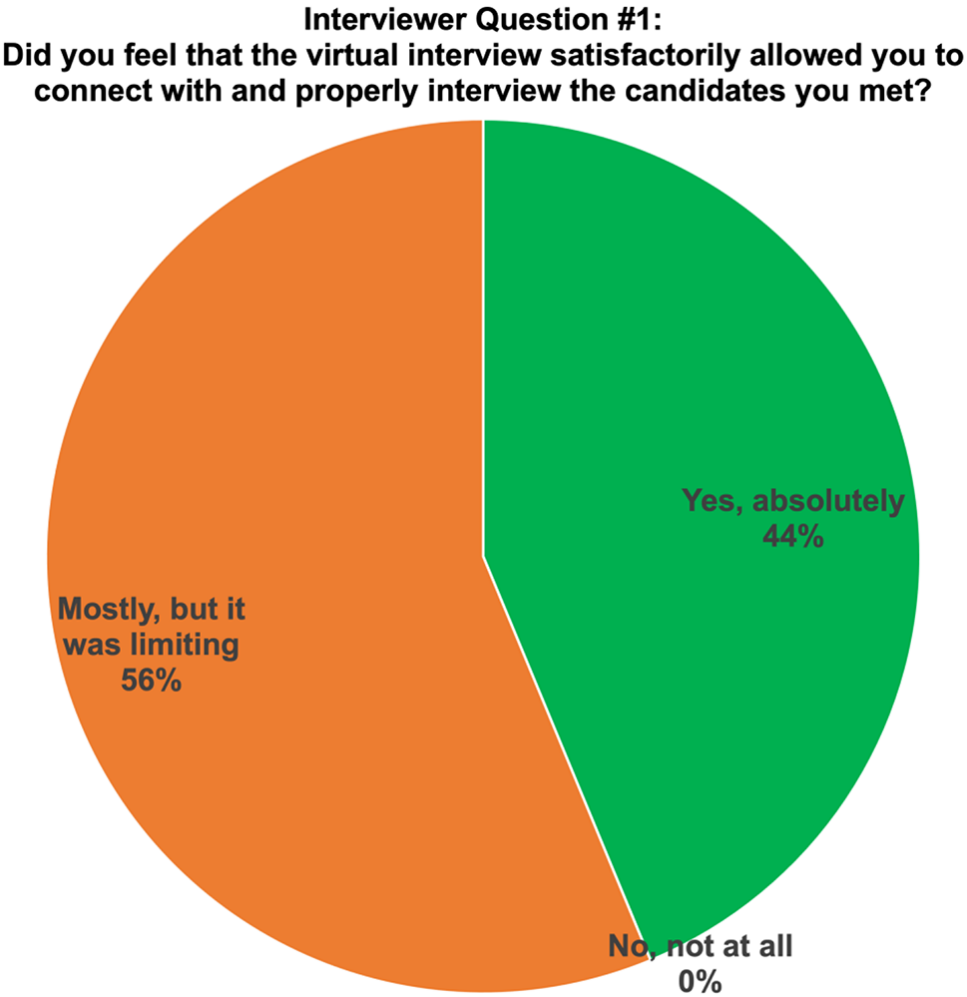
Results from interviewers on the their perspective of using virtual interviews to being able to connect with others

Overall, 68.5% interviewees found that virtual interviewing allowed them to easily talk and connect with others, despite the virtual connection (Fig. 1). Furthermore, 74.1% reported that the virtual interview did not limit their ability to convey their strengths and interests in the program (Fig. 2). Interviewees also found that navigating through virtual interviews was easy. In general, interviewees prefer virtual interviews (27.8%) rather than in-person interviews (16.7%), but most would like to have both options available (55.5%) (Fig. 3). Table 2 displays further interviewees’ experience with virtual interviewing.

**Table 2:** Descriptive analysis of interviewees experience with virtual interviewing

	Frequency (%) *N* = 54
**How was the virtual interview experience in terms of being able to connect with others?**	
I felt it was easy to talk and connect, despite the virtual connection	37 (68.5)
It worked pretty well, although I feel I could have connected more effectively in person	14 (25.9)
Almost half of the session were fine, and the other half were strained	0 (0)
It was very limiting and I fear the physical distancing was prohibitive for me and for my interviewers	2 (3.7)
In most of the sessions, I felt very awkward and worried the other person/people were not getting to know me well because of the virtual format	1 (1.9)
**The virtual interview limited my ability to convey my strengths and interests in the residency program.**	
Strongly disagree	17 (31.5)
Somewhat disagree	23 (42.6)
Neutral	7 (12.9)
Somewhat agree	6 (11.1)
Strongly agree	1 (1.9)
**The virtual interview limited my ability to assess the program’s educational environment and whether I would fit in well.**	
Strongly disagree	6 (11.1)
Somewhat disagree	11 (20.4)
Neutral	11 (20.4)
Somewhat agree	23 (42.6)
Strongly agree	3 (5.5)
**Assuming resolution of the pandemic**,** in your opinion**,** the recruitment process should be**:	
All in-person interviews	9 (16.7)
All virtual interviews	15 (27.8)
Both options available	30 (55.5)
**Was navigating through the format of online interviews difficult?**	
Yes	1 (1.9)
Somewhat	3 (5.5)
No	47 (87.0)
No answer	3 (5.5)

#### Interviewers

**Fig. 4:**
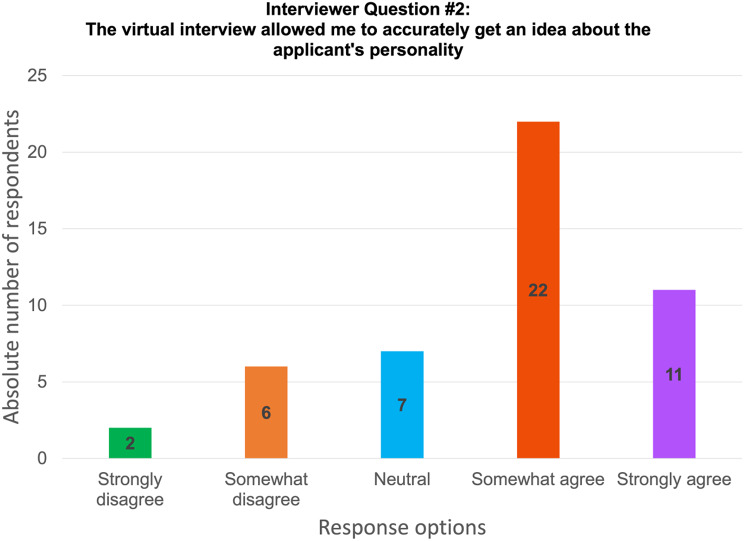
Results from interviewers on their ability to accurately get an idea about the applicant’s personality through virtual interviewing

**Fig. 5:**
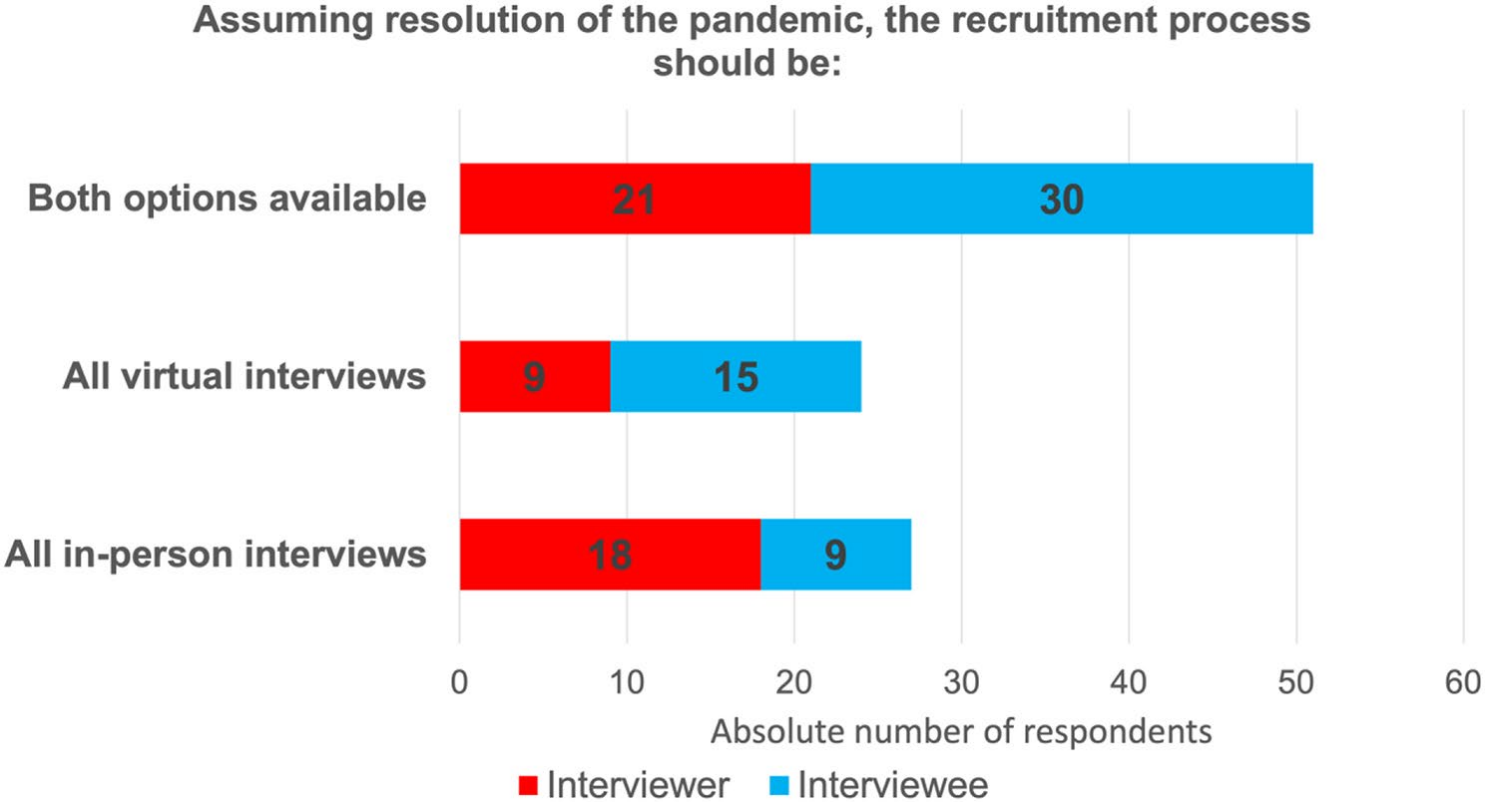
Survey question and data from interviewers and interviewees on their preference of interview format

Overall, virtual interviewing satisfactorily allowed interviewers to connect with and properly interview candidates, but 56.25% of participants found the experience to be limiting in terms of interacting with candidates (Fig. 4). 68.7% of interviewers agreed that they were able to get an idea about the applicant’s personality while 77.1% were comfortable ranking their candidates (Fig. 5). No additional resources (e.g., time, funding) were required as compared to in-person interviewing by 68.5% of interviewers. The interviewers found that navigating through virtual interviews was easy. Interviewers were asked to rate different aspects of virtual interviews i.e., financial burden, time burden and ability to assess an applicant’s fit into the program relative to in-person interviews, with 1 being much less stressful, 5 being more stressful and 3 being equivocal. Financial burden was rated as 3 by 79.2%, time burden was rated as 3 as well by 43.8%. However, ease of access was rated as less stressful by 37.5% of interviewers while their ability to assess an applicant’s fit into the program was rated as 4 or 5 by 52.1%. In general, interviewers prefer in-person interviews (37.8%) rather than virtual interviews (18.75), but most interviewers would rather have both options available (43.75%) (Fig. 3). Table 3 displays further interviewees’ experience with virtual interviewing.

**Table 3:** Descriptive analysis of interviewers’ experience with virtual interviewing

	Frequency (%) *N* = 48
**Did you feel that the virtual interview satisfactorily allowed you to connect with and properly interview the candidates you met?**	
Yes, absolutely	21 (43.75)
Mostly, but it was limiting	27 (56.25)
No, not at all	0 (0)
**Did transitioning to a virtual interview format require any additional resources**,** whether faculty time**,** stop time**,** or funds**,** as compared to the in-person format?**	
Yes	15 (31.25)
No	33 (68.75)
**After experiencing interviews this cycle**,** what would be your preference?**	
All in-person interviews	18 (37.5)
All virtual interviews	9 (18.75)
Both options available	21 (43.75)
**Was navigating through the format of online interviews difficult?**	
Yes	1 (2.1)
Somewhat	13 (27.1)
No	34 (70.8)
**The virtual interview allowed me to accurately get an idea about the applicant’s personality**	
Strongly disagree	2 (4.2)
Somewhat disagree	6 (12.5)
Neutral	7 (14.6)
Somewhat agree	22 (45.8)
Strongly agree	11 (22.9)
**I felt comfortable ranking the candidates based on my video interview**	
Strongly disagree	1 (2.1)
Somewhat disagree	3 (6.3)
Neutral	7 (14.6)
Somewhat agree	23 (47.9)
Strongly agree	14 (29.2)
**How do you think the virtual interview format changed your stress level compared to potential in-person interviews about the following factors? (1 = much less stressful than in-person interviews**,** 5 = much more stressful than in-person interviews)**	
**A) Financial Burden**	
1	4 (8.3)
2	5 (10.4)
3	38 (79.2)
4	1 (2.1)
5	0 (0)
**B) Time Burden**	
1	7 (14.6)
2	8 (16.6)
3	21 (43.8)
4	11 (22.9)
5	1 (2.1)
**C) Ease of Access**	
1	6 (12.5)
2	12 (25.0)
3	15 (31.25)
4	15 (31.25)
5	0 (0)
**D) Ability to assess “fit” and “culture” at program**	
1	0 (0)
2	3 (6.2)
3	20 (41.7)
4	20 (41.7)
5	5 (10.4)

#### Differences between interviewees and interviewers

In regards to virtual interviewing, there were major differences in answers between interviewers and interviewees (Table 4). There was a difference in the distribution of difficulty in navigating through virtual interviews between interviewees and interviewers (*p* = 0.0116). This difference in distribution can also be seen in their preference of interview format (*p* = 0.0005).

**Table 4:** Bivariate analysis of the participants’ responses in relation to virtual interviewing

Interviewer and Interviewee Question	*p*-value
Was navigating through the format of online interviews difficult?	0.0116
After experiencing interviews this cycle, what would be your preference?	0.0005

#### Qualitative findings

Interviewees and interviewers expressed similar *advantages of virtual interviews* such as the reduced cost, time, and environmental burden due to less travelling. Interviewees also found the experience to be less stressful than if they were going to prepare for in-person interviews. In terms *of disadvantages*, interviewees had difficulty in assessing clinical sites and interviewers found it difficult to display their sites. Table 5 summarizes the advantages and disadvantages brought forward by both groups.

**Table 5:** Advantages and disadvantages of virtual interviewing, with illustrative quotations

	Interviewees	Interviewers
Advantages	• Reduced cost, time, and environmental burden “[…] reduction in financial cost, the stress of travelling and the environmental impact I personally feel outweigh the limitations of virtual interviewing.” – P39 • Increased comfort and reduced overall stress “In some ways [virtual interviews] were less stressful as you were doing them at home as opposed to travelling which can be tiring and time consuming.” – P32	• Reduced cost, time, and environmental burden “Virtual interviewing is significantly more convenient due to less expense, less time away and smaller carbon footprint.” – P3
Disadvantages	• Unable to visit clinical sites and meet residents/staff in-person “The virtual site meetings were useful but it would have been more informative to meet the staff/residents and see the sites in person” – P33 • Difficult to demonstrate nonverbal communication “The only drawback is having to fix our camera, which allows the interviewer to have eye contact with the candidate. However, the candidate does not always have the visual on the interviewer because he is concentrating on looking at the camera.” -P46	• Difficult to display location sites “The downside for us as a program is that we have less opportunity to show our sites to non-McGill students to entice them to come.” -P48 • Increased amount of applicant interviews increased interviewer stress and workload • Noticed applicants were not motivated or interested in program “[T]oo many candidates because of the virtual [sic], candidates [were] not very interested in coming to Gatineau and they seem to be doing interviews to practice, I felt like I was wasting my time.” – P38

### Views on emerging technologies

#### Interviewees

**Fig. 6:**
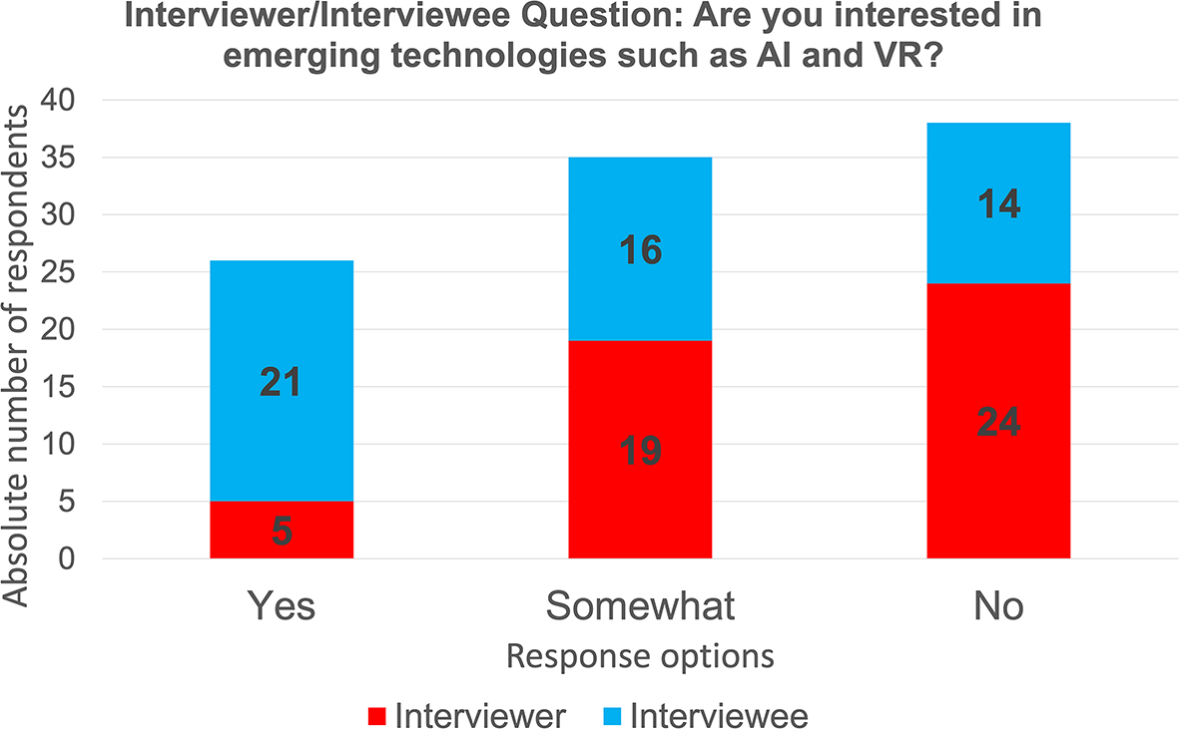
Survey question and data from interviewers and interviewees on their interest in emerging technologies

**Fig. 7:**
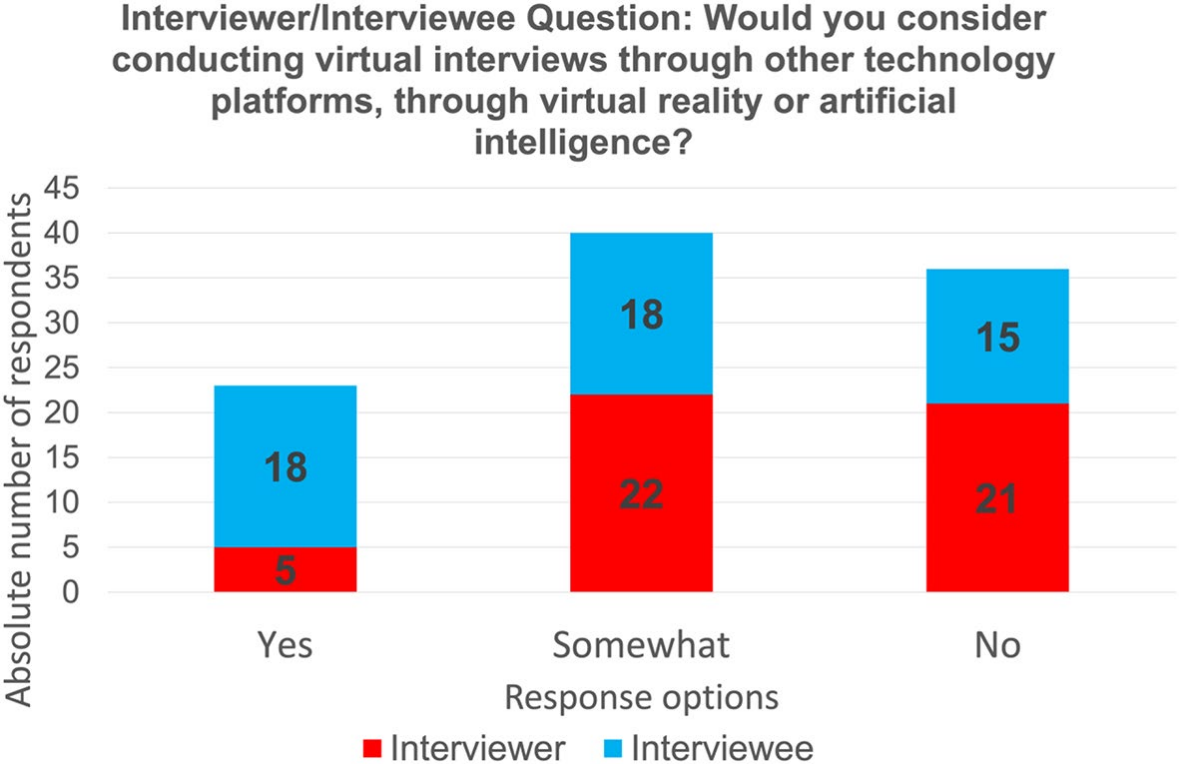
Survey question and data from interviewers and interviewees on ever considering conducting virtual interviewers with VR or AI

Seventy-two per cent (72%) of the interviewees were interested in emerging technologies (Fig. 6). In terms of adopting these technologies into virtual interviewing, 75% would at least consider it if given the chance (Fig. 7). Interviewees were indifferent in terms of trust and reliability for virtual interviewing (43.8%) and clinical practice (43.8%). Many interviewees (95.8%) were interested in learning about AI and VR and its application in clinical practice. Majority (60.8%) agreed that it should be taught within medical training. Only one participant completed a previous course in emerging technologies, specifically focusing on handling personal data using AI. For those who did not complete a course in emerging technologies, 82.3% of interviewees would be interested in taking a course on both AI and VR while 5.9% would only be interested in taking a course only for AI (Table 6).

**Table 6:** Descriptive analysis of interviewees experience with emerging technologies

	Frequency (%) *N* = 51
**Are you interested in emerging technologies such as AI and VR?**	
Yes	21 (41.2)
Somewhat	16 (31.4)
No	14 (27.4)
**Would you ever consider conducting virtual interviews through other technology platforms**,** through virtual reality or artificial intelligence?**	
Yes	18 (35.3)
Somewhat	18 (35.3)
No	15 (29.4)
**Respond to the following statement: I find emerging technologies to be trustable and reliable in virtual interviews**	
Strongly disagree	2 (3.9)
Somewhat disagree	6 (11.8)
Neutral	21 (41.2)
Somewhat agree	16 (31.4)
Strongly agree	6 (11.8)
**Respond to the following statement: I find emerging technologies to be trustable and reliable in clinical practice**,** specifically in family medicine**	
Strongly disagree	1 (2.0)
Somewhat disagree	9 (17.6)
Neutral	21 (41.2)
Somewhat agree	17 (33.3)
Strongly agree	3 (5.9)
**Respond to the following statement: I believe family residents should learn AI**,** VR related concepts within medical training.**	
Strongly disagree	0 (0)
Somewhat disagree	5 (9.8)
Neutral	15 (29.4)
Somewhat agree	23 (45.1)
Strongly agree	8 (15.7)
**Would you be interested in learning about AI**,** VR and its application in family medicine practice?**	
Yes	46 (90.2)
No	5 (9.8)
**Did you complete a course where AI**,** VR was being taught?**	
Yes	1 (2.0)
No	50 (98.0)
**Are you willing to take a course where AI or VR or both is taught?**	
Yes for AI	3 (5.9)
Yes for VR	0 (0)
Yes for both	42 (82.3)
No	5 (9.8)
I have already taken a course in AI or VR	1 (2.0)

#### Interviewers

Half of the interviewers were interested in emerging technologies (Fig. 6). In terms of adopting these technologies into virtual interviewing, 56.3% would at least consider it if given the chance, but 54.2% of interviewers were not comfortable using it at that moment (Fig. 7). Interviewers were indifferent in terms of trust and reliability of emerging technologies for virtual interviewing (60.4%) and applying it in clinical practice (43.8%). Although interviewers were indifferent in terms of trust and reliability of these technologies, 81.3% would not consider using these technologies for either virtual interviewing or clinical practice (Table 7).

**Table 7:** Descriptive analysis of interviewers experience with emerging technologies

	Frequency (%) *N* = 48
**Are you interested in emerging technologies such as AI and VR?**	
Yes	5 (10.4)
Somewhat	19 (39.6)
No	24 (50.0)
**Would you ever consider conducting virtual interviews through other technology platforms**,** through virtual reality or artificial intelligence?**	
Yes	5 (10.4)
Somewhat	22 (45.8)
No	21 (43.8)
**How comfortable would you feel integrating emerging technology during virtual interviews?**	
Uncomfortable	26 (54.2)
Somewhat comfortable	18 (37.5)
Very comfortable	4 (8.3)
**Respond to the following statement: I find emerging technologies to be trustable and reliable in virtual interviews**	
Strongly disagree	2 (4.2)
Somewhat disagree	10 (20.8)
Neutral	29 (60.4)
Somewhat agree	7 (14.6)
Strongly agree	0 (0)
**Respond to the following statement: I find emerging technologies to be trustable and reliable in clinical practice**,** specifically in family medicine**	
Strongly disagree	4 (8.3)
Somewhat disagree	13 (27.1)
Neutral	21 (43.8)
Somewhat agree	10 (20.8)
Strongly agree	0 (0)
**Would you consider using emerging technologies for teaching and/or evaluating residents?**	
Yes	26 (54.2)
No	22 (45.8)
**Do you feel ready to use emerging technologies within interviews or clinical practice?**	
Yes	9 (18.7)
No	39 (81.3)

#### Differences between interviewees and interviewers

In regards to emerging technologies, significant differences of answers between interviewers and interviewees were also seen (Table 8). There were differences between interviewees and interviewers in terms of participants’ interest in AI/VR (*p* = 0.00179), their consideration of using AI or VR for virtual interviewing (*p* = 0.0131) and their trust in using these technologies for virtual interviews (*p* = 0.0196). However, in terms of their trust in using emerging technologies for clinical practice, there was no significant difference (*p* = 0.1229) between the two groups.

**Table 8:** Bivariate analysis of the participants’ responses in relation to emerging technologies

Interviewer and Interviewee Question	*p*-value
Are you interested in emerging technologies such as AI and VR?	0.00179
Would you ever consider conducting virtual interviews through other technology platforms, through virtual reality or artificial intelligence?	0.0131
Respond to the following statement: I find emerging technologies to be trustable and reliable in virtual interviews	0.0196
Respond to the following statement: I find emerging technologies to be trustable and reliable in clinical practice, specifically in family medicine	0.1229

#### Qualitative findings

Interviewers had no knowledge or experience of AI or VR for recruitment and education, but little to no experience within clinical practice (e.g., scheduling, and patient communication). Of these three domains, interviewers were most excited for AI and VR for teaching and evaluation of residents. One participant mentioned potential uses such as, “simulations, practice exams, demonstrations of clinical exams, supervision when the boss cannot be physically present” (P28). Interviewers were open to the possibility of emerging technologies in recruitment and clinical practice but were nonetheless hesitant. Table 9 summarizes interviewers’ opinion on using emerging technologies within the three domains of recruitment, education, and clinical practice.

**Table 9:** Interviewers’ opinions on using emerging technologies for recruitment, education and clinical practice, with illustrative quotations

Recruitment (Virtual Interviews)	Education (Teaching and Evaluation)	Clinical Practice
• No knowledge or experience • Open to trying, but hesitant due to the added level of complexity of the virtual format	• No knowledge or experience • Excited for its use in “simulations, practice exams, demonstrations of clinical exams, supervision when the boss cannot be physically present” – P28	• Little to no experience • Training required and added support to develop comfort • Hesitant due to busy schedule and bias associated with AI

## Discussion

In postgraduate medical education, the interview process is an essential component for applicants and programs alike [23]. For students, their residency training program and site have a great impact on their career and life, while residency program directors/faculty seek out new residents who are a good fit for their program. ‘A good fit’ usually includes a strong academic performance, well developed interpersonal skills, and a positive attitude to the specialty and learning [2]. The COVID-19 pandemic forced several residency programs to adapt to a new virtual interview format, and thus navigate through new technologies. The role of technology in medical education such as videoconferencing tools are vital, especially during times of rapid and unknown change. As medicine continues to advance, further problems are introduced, and thus innovative solutions are needed such as AI and VR in order for amelioration.

### Virtual interviews

Conceived to document the experience of participating in virtual interviews for recruiting family medicine residents during the first two years of the COVID-19 pandemic, the first major finding of this investigation is that the virtual interview process was satisfactory for both groups of participants, and the interview process ran with ease. This finding further corroborates the literature for family medicine applicants and program directors experience with virtual interviews [24, 25]. Other studies in different specialities such as neurosurgery and surgical oncology have also reported similar findings [26, 27].

A second major finding of this study is that, according to participants, virtual interviewing implies important advantages vis-à-vis in-person interviews, notably a reduction in cost, time, and environmental burden. This is seen as both groups do not have to travel to the site to conduct the interview, which reduces costs on all three of the abovementioned aspects. This finding is also consistent with prior evidence, that indicates how virtual interviews might be cost-effective compared to in-person interviews [28].

Likewise, our study also corroborates the results obtained in prior investigations that pointed out several disadvantages of virtual interviewing for recruiting medical residents such as technical challenges, lack of personal connection between applicants and faculty, and increased difficulty for the applicant to view the culture of the program, the hospital campus, and the city in-person [23]. In addition, interviewer respondents reported an increase in applicants during the virtual format, compared to previous recruitment cycles. This may be due to fewer cancellation and declines, resulting in an inflation of applicants [14, 29].

Videoconferencing technology such as Zoom platform was seen as inferior to meeting a person in a room. The interviewer and interviewee are partially visible, the screen displaying the individual is smaller than in real life, and the quality of the sound and image is generally inferior to an in-person visit. Concerns were raised about interviewers’ ability to appreciate and notice nonverbal communication. In-person recruitment and selection activities often coupled short individual interviews with longer interactive activities that demonstrated interpersonal skills such as the positive and respectful interaction with other applicants, staff, or interviewers. These interactions may be missed in time and place limited online interviews or orientation.

In this study, interviewers (43.75%) and interviewees (55.5%) would prefer for both options to be available once the pandemic restrictions were no longer necessary. Importantly, both groups noted that the virtual option should only be used for applicants who are restricted due to location. In addition, interviewees recommended that program directors/faculty should utilize virtual components during the process such as virtual site meetings or virtual information sessions. Whereas some authors argue that virtual interviews should prevail, with some optional in-person events implemented [12, 14, 32], our suggested position has also been adopted in other medical specialties (e.g., pediatric, and general surgery) in which a virtual component should be utilized in addition to in-person interviews instead of a complete replacement [30, 31]. The verdict of which option should remain does therefore vary in the literature with some supporting the transition back to in-person interviews [32–35] and other preferring utilizing virtual interviews instead [5].

### Emerging technology

There has been a rise of the use of information technologies such as videoconferencing tools [15], as well as emerging technologies such as AI and VR, in both medical education [36] and clinical practice. AI has emerged as a powerful tool in medical education, facilitating personalized learning, adaptive assessments, and advanced analytics to optimize the educational process [37]. Moreover, AI-driven diagnostic tools and decision support systems are gaining traction in clinical practice, assisting healthcare professionals in making more accurate and timely decisions [38] Similarly, VR has revolutionized medical education by offering immersive and interactive training experiences. Medical students and professionals can now engage in realistic simulations to hone their skills and practice complex procedures in a safe and controlled environment [39, 40]. Furthermore, VR has found applications in clinical practice, notably in areas such as pain management, rehabilitation, and mental health treatment [41].

The perception of emerging technology is considerably different between our study’s two groups. The findings of our investigation strongly suggest that applicants are much keener to learn about and use emerging technologies compared to interviewers. This may be a generational gap in comfort, trust, and experience with emerging technology [42]. Applicants expressed a need to integrate AI education (e.g., knowledge and skills related to AI applications) within their training. This need has been echoed from other future physicians who agree that there should be an update on the current medical curriculum with the addition of AI topics [43]. However, interviewers who are current family physicians and family medicine educators were less enthusiastic about the integration of technologies within both medical education and clinical practice. They were skeptical about the impact of technology, specifically AI, on improving the quality of clinical practice. This sentiment has also been shared by British general practitioners who are doubtful that technology will perform most primary care tasks as well as or better than human physicians [44]. Further views concerning AI in primary care were seen by primary health care and digital health stakeholders in Ontario who have a hopeful, but guarded stance as there are several factors that must considered [45]. Our study demonstrates the polarizing difference in opinion of emerging technology for interviewees and interviewers; however both groups do agree that AI and VR will increasingly expand in medicine, especially within their speciality.

### Limitation

Our study has a number of limitations, which mainly concern the research design adopted, which allows capturing data at a single moment in time. Moreover, although data was collected for both types of participants and over two recruitment cycles, the study was conducted in a single academic institution of a developed country. Hence, one should be cautious when generalizing the lessons learnt to other medical schools and residency programs as it might not echo the evolving patterns or shifts in the field. In addition, we analyzed participants’ self-reported data, which could be subject to response bias as participants, specifically interviewees may have felt pressure to respond positively. Moreover, heterogeneity between the education level and background of both interviewers and interviewees was observed, which could have further influenced their opinions and answers. Self-reported data may also result in potential recall bias for interviewees that were from the 2020–2021 cycle compared to the 2021–2022 cycle. To reduce this bias as much as possible, the survey was distributed after all interviews were completed and matched for the 2021–2022 cycle. Non-response bias was also minimized throughout this study as the survey was brief and participants were reminded after two and four weeks. Future research should consider incorporating a broader range of academic institutions and specialties, as well as using alternative data collection methods to further validate and expand upon our findings.

## Conclusion

The transition to virtual interviews for family medicine residency recruitment was both prompt and unexpected during the COVID-19 pandemic. However, most interviewers and interviewees reported positive experiences with virtual interviews. Although there are aspects that could be better assessed through in-person interviews, there is consensus about the benefits of virtual interviews such as decreased cost and time. Overall, it is unlikely that virtual interviews will completely replace in-person interviews for selecting candidates for family medicine residency programs in the long term as participants value aspects of in-person interviews and would want a choice in format. However, it is important to understand current cycles’ experience to improve future residency application processes, albeit virtual or in-person.

Additionally, the apparent eagerness of incoming family medicine doctors to learn and adopt cutting-edge technologies suggests that educators and institutions should foresee and meet the needs of practicing family doctors and residents in two crucial areas: (1) AI and VR education, and (2) the provision of resources to support the integration of AI/VR into clinical practice. It is important to comprehend the needs and expectations of future family medicine residents. Institutions can better adjust their interview procedures and training programs to take advantage of cutting-edge technologies and meet the changing demands of aspiring healthcare professionals by studying the preferences and expectations of prospective residents. Although this study involved only one institution and speciality, the information gathered may be useful for other institutions with a family medicine residency program.

## Electronic supplementary material

Below is the link to the electronic supplementary material.

Supplementary Material 1

## Data Availability

All data generated or analysed during this study are included in this published article and its supplementary information files.

## Abbreviations

AI: Artificial intelligence
AFMC: Association of faculties of medicine of canada
COVID-19: Coronavirus disease of 2019
CROSS: Checklist for reporting of survey studies
VR: Virtual reality
