# Supplementary material for: Perspectives on virtual interviews and emerging technologies integration in family medicine residency programs: a cross-sectional survey study
Source: BMC Med Educ. 2024 Sep 9;24:975. doi: 10.1186/s12909-024-05874-5 (PMC11382399; doi:10.1186/s12909-024-05874-5)
Supplement: Supplementary file 1 — Supplementary Material 1 [file 12909_2024_5874_MOESM1_ESM.docx]

**BMC Medical Education - Supplementary Material**

**Appendix 1**: **General Information (Sociodemographic) for Interviewers and Interviewees**

1. **In terms of sex, how do you identify?**
   1. Male
   2. Female
   3. I prefer not to answer
   4. Other
2. **In terms of gender, how do you identify yourself?**
   1. Male
   2. Female
   3. I prefer not to answer
   4. Other
3. **What is your age?**
   1. ≤25 years old
   2. 26-30 years old
   3. 31-35 years old
   4. 36-40 years old
   5. ≥ 41 years old
   6. I prefer not to answer
4. **Which category best describes you?**
   1. White or Caucasian
   2. Black or African American
   3. Asian or Pacific Islander
   4. Hispanic or Latinx
   5. Multiracial or Biracial
   6. Indigenous
   7. I prefer not to answer
   8. Other
5. **What is your primary language?**
   1. English
   2. French
   3. Other
6. **What is you highest level of education before/after MD?**
   1. Bachelor’s
   2. Master’s
   3. Doctorate
   4. Other
7. **What is your educational background?**
   1. Biomedical Science and Health Sciences
   2. Arts and Humanities
   3. Business
   4. Engineering, Math and Computer Science
   5. Other

**Appendix 2: Questions for Faculty (Interviewers)**

*Appendix 2AQuestions about virtual interviews for interviewers*

1. **Did you feel that the virtual interface satisfactorily allowed you to connect with and properly interview the candidates you met**
   1. Yes, absolutely
   2. Mostly, but it was limiting
   3. No, not at all
2. **Did transitioning to a virtual interview format require any additional resources, whether faculty time, stop time, or funds, as compared to the in-person format?**
   1. Yes
   2. No
3. **After experiencing interviews this cycle, what would be your preference? Why? Please write your following explanation in the space provided.**
   1. All in-person interviews
   2. All virtual interviews
   3. Both options available
4. **Respond to the following statement: The virtual interview allowed me to accurately get an idea about the applicant's personality**
   1. Strongly disagree
   2. Somewhat disagree
   3. Neutral
   4. Somewhat agree
   5. Strongly agree
5. **Respond to the following statement: I felt comfortable ranking the candidates based on my video interview**
   1. Strongly disagree
   2. Somewhat disagree
   3. Neutral
   4. Somewhat agree
   5. Strongly agree
6. **How do you think the virtual interview format changed your stress level compared to potential in-person interviews about the following factors? A) Financial Burden**
   1. 1 = much less stressful than in-person interviews
   2. 2 = less stressful than in-person interviews
   3. 3 = no difference in stress level
   4. 4 = more stressful than in-person interviews
   5. 5 = much more stressful than in-person interviews
7. **How do you think the virtual interview format changed your stress level compared to potential in-person interviews about the following factors? B) Time Burden**
   1. 1 = much less stressful than in-person interviews
   2. 2 = less stressful than in-person interviews
   3. 3 = no difference in stress level
   4. 4 = more stressful than in-person interviews
   5. 5 = much more stressful than in-person interviews
8. **How do you think the virtual interview format changed your stress level compared to potential in-person interviews about the following factors? C) Ease of Access**
   1. 1 = much less stressful than in-person interviews
   2. 2 = less stressful than in-person interviews
   3. 3 = no difference in stress level
   4. 4 = more stressful than in-person interviews
   5. 5 = much more stressful than in-person interviews
9. **How do you think the virtual interview format changed your stress level compared to potential in-person interviews about the following factors? D) Ability to assess “fit” & “culture” at program**
   1. 1 = much less stressful than in-person interviews
   2. 2 = less stressful than in-person interviews
   3. 3 = no difference in stress level
   4. 4 = more stressful than in-person interviews
   5. 5 = much more stressful than in-person interviews
10. **What aspects of virtual interviewing are you most likely to retain or change moving forward, regardless of whether virtual recruitment continues? Please write your answer below.**
11. **Additional comments on virtual interviewing? Please write your answer below.**

*Appendix 2B: Questions about novel technology for interviewers*

1. **Was navigating through the format of online interviews difficult?**
   1. Yes
   2. Somewhat
   3. No
2. **Are you interested in novel technologies such as AI and VR? Virtual reality (VR) can defined as a platform for simulation or replication of certain digitally constructed environments. Artificial intelligence (AI) is defined as “the performance by computer programs of tasks that are commonly associated with intelligent beings” by the World Health Organization.**
   1. Yes
   2. Somewhat
   3. No
3. **Would you ever consider conducting virtual interviews through other technology platforms, through virtual reality or artificial intelligence? For example, the interviewee and interviewer, who may be in separate locations, utilizes a head mounted display to enter a virtual platform (ex. MeetinVR) where they are able to meet one another such as in a virtual room. The individuals would then be able to speak and interact with each other just as they would in real life.**
   1. Yes
   2. Somewhat
   3. No
4. **How comfortable would you feel integrating AI technologies or VR during virtual interviews? Why?** **Please write your following explanation in the space provided.**
   1. Very comfortable
   2. Somewhat comfortable
   3. Uncomfortable
5. **Respond to the following statement: I find AI and VR technology to be trustable and reliable in virtual interviews**
   1. Strongly disagree
   2. Somewhat disagree
   3. Neutral
   4. Somewhat agree
   5. Strongly agree
6. **Respond to the following statement: I find AI and VR technology to be trustable and reliable in clinical practice, specifically in family medicine**
   1. Strongly disagree
   2. Somewhat disagree
   3. Neutral
   4. Somewhat agree
   5. Strongly agree
7. **Would you consider using novel technology (e.g., AI, VR) for teaching and/or evaluating residents? If yes, how would you use these novel technology (e.g., AI, VR) for teaching and/or evaluation of residents? Please write your following explanation in the space provided.**
   1. Yes
   2. No
8. **Do you feel ready to use novel technology (e.g., AI, VR) within interviews or clinical practice? Why? In addition, what resources/supports will you need? Please write your following explanation in the space provided.**
   1. Yes
   2. No
9. **Would you like to be contacted for other studies investigating AI, VR within family medicine? If yes, please provide your name and email to be contacted in the future.**

**Appendix 3: Questions for Applicants (Interviewees)**

*Appendix 3A: Questions about virtual interviews for interviewees*

1. **How was the virtual interface for you overall, as far as being able to connect with others?**
   1. I felt it was east to talk and connect, despite the virtual connection
   2. It worked pretty well, although I feel I could have connected more effectively in person
   3. Almost half of the session were fine, and the other half were strained
   4. It was very limiting and I fear the physical distancing was prohibitive for me and for my interviewers
   5. In most of the sessions, I felt very awkward and worried the other person/people were not getting to know me well because of the screen interface
2. **Respond to the following statement: The virtual interface during interviews limited my ability to convey my strengths and interest in your program**
   1. Strongly disagree
   2. Somewhat disagree
   3. Neutral
   4. Somewhat agree
   5. Strongly agree
3. **Respond to the following statement: The virtual interface during interviews limited my ability to assess program’s educational environment and whether I would fit in well**
   1. Strongly disagree
   2. Somewhat disagree
   3. Neutral
   4. Somewhat agree
   5. Strongly agree
4. **Which of the following interview day factors impacted your decision for Family Medicine?**
   1. Interaction with faculty
   2. Interaction with residents
   3. Orientation sessions
   4. Site virtual meetings
   5. Discussion with other candidates (through chat or in question periods)
   6. Communication from the program
   7. Organization leading up the interview experience and following interview day
   8. Response time to questions and inquiries from the program representatives
5. **Assuming resolution of the pandemic, would you prefer recruitment be virtual or in person for future years?**
   1. In-person interviews
   2. Virtual interviews
   3. Both options available
6. **What aspects of virtual interviewing are you most likely to retain or change moving forward, regardless of whether virtual recruitment continues? Please write your answer below.**
7. **Additional comments on virtual interviewing? Please write your answer below.**

*Appendix 3B: Questions about novel technology for interviewees*

1. **Was navigating through the format of online interviews difficult?**
   1. Yes
   2. Somewhat
   3. No
2. **Are you interested in novel technologies such as AI and VR? Virtual reality (VR) can defined as a platform for simulation or replication of certain digitally constructed environments. Artificial intelligence (AI) is defined as “the performance by computer programs of tasks that are commonly associated with intelligent beings” by the World Health Organization.**
   1. Yes
   2. Somewhat
   3. No
3. **Would you ever consider conducting virtual interviews through other technology platforms, through virtual reality or artificial intelligence? For example, the interviewee and interviewer, who may be in separate locations, utilizes a head mounted display to enter a virtual platform (ex. MeetinVR) where they are able to meet one another such as in a virtual room. The individuals would then be able to speak and interact with each other just as they would in real life.**
   1. Yes
   2. Somewhat
   3. No
4. **Respond to the following statement: I find novel technology (e.g., AI, VR) to be trustable and reliable in virtual interviews**
   1. Strongly disagree
   2. Somewhat disagree
   3. Neutral
   4. Somewhat agree
   5. Strongly agree
5. **Respond to the following statement: I find novel technology (e.g., AI, VR) to be trustable and reliable in clinical practice, specifically in family medicine**
   1. Strongly disagree
   2. Somewhat disagree
   3. Neutral
   4. Somewhat agree
   5. Strongly agree
6. **Would you be interested in learning about AI, VR in family medicine and their associated applications?**
   1. Yes
   2. No
7. **Did you complete a course where AI, VR was being taught? If yes, please explain more about the course and your experience. Please write your following explanation in the space provided.**
   1. Yes
   2. No
8. **If your answer to the previous question was no, are you willing to take a course where AI or VR or both is taught?**
   1. Yes for AI
   2. Yes for VR
   3. Yes for both
   4. No
   5. I have already taken a course in AI or VR
9. **Respond to the following statement: I believe family residents should learn AI, VR related concepts within medical training.**
   1. Strongly disagree
   2. Somewhat disagree
   3. Neutral
   4. Somewhat agree
   5. Strongly agree
10. **Would you like to be contacted for other studies investigating AI, VR within family medicine? If yes, please provide your name and email to be contacted in the future.**
    1. Yes
    2. No
